# NHS reforms in England: the implications for chemotherapy commissioning

**DOI:** 10.3332/ecancer.2014.451

**Published:** 2014-08-05

**Authors:** Carolyn Staines, Cathy Wright, Janis Smy

**Affiliations:** Succinct Medical Communications, Marlow, Buckinghamshire SL7 1AB, UK

**Keywords:** Cancer Drugs Fund, Clinical Commissioning Groups, Clinical Reference Groups, NHS England, systemic anticancer treatment, value-based assessment

## Abstract

For oncology, one of the biggest effects of the reforms to the National Health Service (NHS) in England has been the designation of systemic anticancer treatments (other than hormonal agents) as a specialised service. This means that all decisions regarding the commissioning of chemotherapy are made at the national level via NHS England (NHSE), under expert guidance from the Chemotherapy Clinical Reference Group (CRG). Commissioning decisions will be based on several factors, not only clinical efficacy and cost/affordability, but also the ‘added value’ that a new treatment may offer in terms of patient outcomes, resource utilisation, and/or wider benefits to society. Oncology health-care professionals (HCPs) are in a position to affect cancer commissioning decisions in the reformed NHS, not only the small number who are members of the Chemotherapy CRG, but also as advisors to the Chemotherapy CRG via disease-specific consensus work, and through participation in the collection and reporting of real-life data on patient outcomes. With the emerging emphasis on both consensus work and outcomes data, a step change can also be expected in the relationship between HCPs and the pharmaceutical industry, including a strengthened role for non-promotional education, support for forums and consensus groups, and facilitation of the development, collection, and dissemination of findings from real-life practice. In addition, there will be an onus on the pharmaceutical industry to provide information on the implications that new products may have for service delivery and capacity and for meeting patients’ and society’s expectations. This information will need to be developed and delivered in a timely way, well in advance of the launch of a new product.

## Introduction

The National Health Service (NHS) in England has undergone a process of reform that many regard as the biggest upheaval in the organisation since it was founded in 1948. Root-and-branch changes outlined initially in the Health and Social Care Act 2012 largely came into effect in April 2013, with the abolition of long-established NHS structures, and the creation of brand new structures and decision-making processes [1]. Many of the new processes, including those for commissioning medicines, continue to evolve. Some of the changes are still shrouded in uncertainty—plans for value-based assessment of pharmaceutical agents being a notable example [2]. Fundamental to the reforms is a single operating procedure and national model for the commissioning of certain specialised treatments, including chemotherapy and mechanisms for engaging health-care professionals (HCPs) in the evaluation and assessment of medications [3]. The reforms also place a strong emphasis on achieving increased value for NHS money [4].

The rationale for the reforms and the workings of the various new organisations and processes are set out in detail in an array of freely available government documents. However, as specialists in medical communications and education, with a strong focus on oncology, we have become aware that many HCPs in the frontline of cancer care are perplexed by the new commissioning environment, unclear about the implications for the ongoing provision of effective chemotherapy, and uncertain about the form of support that can usefully be provided by the pharmaceutical industry. Hence, this article considers the mechanisms now in place, and those that may soon develop, for commissioning treatments in the reformed NHS in England, the pathways of particular relevance to chemotherapy agents, the emerging new platforms for interaction between NHS HCPs and the pharmaceutical industry, and some of the uncertainties that still require clarification. Given the dynamic nature of the reforms, this article necessarily provides a “snapshot” view of the ongoing and predicted issues as they appear at the time of writing in March 2014.

## Commissioning in England’s new NHS

A large proportion of responsibility for the NHS in England has passed from the Department of Health to the newly formed strategic body NHS England (NHSE), which reports to the Secretary of State for Health [1].

**Figure 1.**
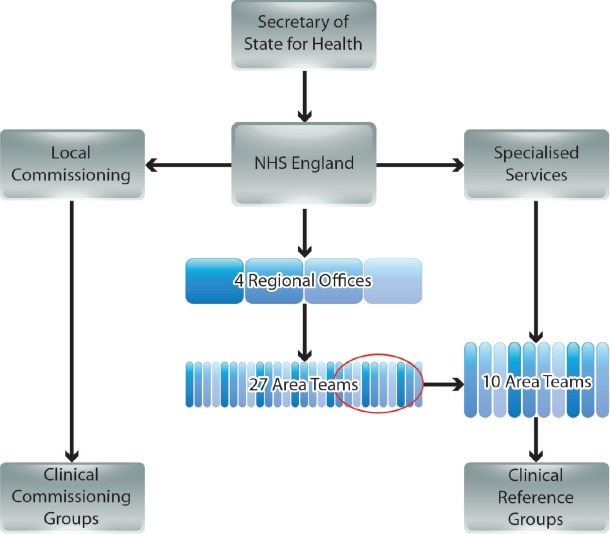
The new NHS commissioning environment in England (simplified from government documents).

NHSE operates at national level with its strategic decisions cascaded via four regional offices (covering North of England, Midlands and East of England, London, and South of England), and managed by 27 Area Teams (Figure 1) [3, 5]. The commissioning of most services is the responsibility of 211 Clinical Commissioning Groups (CCGs), each a collaboration of neighbouring general practices [3]. However, commissioning of certain specialised services is conducted nationally by NHSE, supported by ten of the 27 Area Teams [6]. Expert guidance on each of the specialised services is provided by national disease-specific CRGs, which comprise a mixture of stakeholders, including HCPs, patients, and carers [6–8].

The CRGs have the responsibility for devising national policies for commissioning treatments [8]. This includes determining the criteria for selection or otherwise of individual medicines and, crucially, the ongoing development of national treatment algorithms/pathways. These algorithms are intended to chart patient care from initial diagnosis through multiple lines of intervention, and specify the drugs available for NHS use at each intervention point. When considering the potential use of a new treatment by the NHS, it is expected that CRGs will look at its proposed position on the existing algorithm, and take into account of how its affordability and clinical/resource utilisation outcomes compared with the agents already specified for that stage of patient care.

In April 2014, it was anticipated that the specialised services budget will be overspent to the tune of £336 million [9], and savings will need to be made. Oncology services will be expected to contribute to these savings.

## The chemotherapy perspective

### Commissioning processes

The management of common cancers, such as breast, colon/rectum and lung, is not, *per se*, designated a specialised service; however, chemotherapy for all cancers is commissioned nationally by NHSE, via advice from the Chemotherapy CRG [6–8]. In fact, the Chemotherapy CRG remit covers not only chemotherapy, but also all systemic anticancer treatments (SACTs; excluding hormonal treatments). Using breast cancer as an example, CCGs and CRGs will have input into different aspects of patient care, together forming one large, unified system covering the entire pathway for the disease.

**Figure 2.**
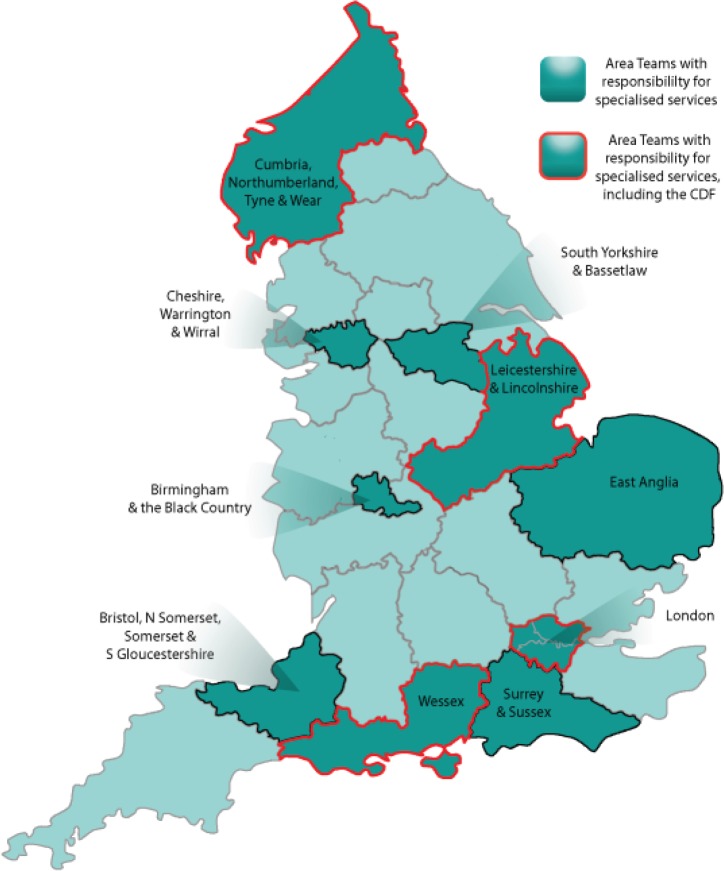
Locations of the 27 Area Teams, highlighting the ten responsible for specialised services, including those that also manage the CDF [6, 10].

In addition to the structures for specialised services discussed earlier, there is one aspect unique to oncology, namely the Cancer Drugs Fund (CDF), which provides patients with access to certain treatments for specific indications where routine NHS funding is not available [11]. Indeed, in October 2010, the newly launched CDF—in interim form—provided oncology HCPs and their patients in England with one of the first manifestations of the forthcoming NHS reforms. The first full-year CDF got underway in April 2011. This ring-fenced fund providing £200 million per annum was initially organised at regional level, and was then re-launched as a single national entity in April 2013 [11], in line with the larger-scale reforms. The CDF was intended to come to an end soon after the implementation of the NHS reorganisation; it has now been extended to at least 2016 [9], and has been absorbed into England’s new policies and structures as a subcommittee of the Chemotherapy CRG [6, 12]. However, it has been suggested that the CDF will require an additional £120 million per year to meet the projected demand [9]. Of the ten Area Teams earmarked for specialised services, four (one per region) have particular responsibility for the administration of the CDF (Figure 2).

NHSE will reimburse provider trusts for the use of SACTs regardless of whether the agent in question has been approved for baseline commissioning (e.g. following a positive health technology appraisal by the National Institute for Health and Care Excellence [NICE]), or if it is approved for use via the CDF.

As well as commissioning SACTs, NHSE is responsible for reimbursing providers for their administration through the national chemotherapy delivery tariffs.

### Decision drivers

While the need to identify cost efficiencies in cancer treatment will be a driver for decision making on the introduction of new SACTs, there is also a duty to consider broader NHS policies and priorities. For example, the NHS Outcomes Framework requires health-care providers to focus on such issues as patient quality of life, treatment side effects, and the avoidance of unplanned hospital admissions for the management of treatment-related ill health [13]. The five domains of the framework and examples of their possible implications for the commissioning of cancer treatments and services are outlined in Table 1. The NHS Mandate requires care for patients with complex needs to be delivered in a way that is centred on the needs of the individual [14]. The NHS Constitution requires a focus on the patient experience [15]. To address such policies and priorities, the Chemotherapy CRG may need to consider models of service delivery based on access to interventions that are not necessarily the cheapest option for achieving defined clinical objectives (e.g., efficacy and safety). Hence, a treatment that can be self-administered by the patient, at home, may be seen to offer added value in terms of use of NHS resources over a lower-cost agent that requires regular attendance at an NHS facility. Even if they cannot avoid attending the chemotherapy unit, patients may prefer to receive an oral or subcutaneous formulation that can be delivered in minutes, rather than have a prolonged intravenous infusion. In these examples, the resource utility of providing care in the NHS facility—in both financial and capacity terms—would also be a consideration for the Chemotherapy CRG.

Of course, cost and affordability remain key issues for any finitely resourced health service—particularly given the projected overspend on specialised services [9]. It is reasonable to assume, therefore, that to gain entry to an established treatment algorithm, a new drug will need to be cost-neutral, or else offer resource utility or net cost savings, compared with the agents already commissioned and offering equal or greater clinical efficacy at the equivalent position in the treatment algorithm.

To meet all of these requirements, commissioning decisions initially based on clinical trial outcomes may need to be validated by real-life data. Indeed, pharmaceutical companies may decide to support real-life studies, outside of clinical trials, as part of the value dossier for assessment and commissioning processes. Real-life outcomes will also be monitored through data collection and analysis by the SACT dataset, which is mandatory from 1 April 2014. Should real-life experience fail to generate the anticipated outcomes, the drug may no longer be affordable. At the very least, such a finding may initiate investigation into the reasons why the predicted outcomes are not being achieved and identify how these issues may be addressed.

## NHS/industry engagement

With regard to new treatments, key roles for HCPs will include (i) horizon scanning for emerging new treatments, (ii) ongoing examination and appraisal of the emerging evidence base, (iii) participation in trials and studies designed to generate real-life data, (iv) identification of the advantages/disadvantages of a new agent in terms of service delivery, and (v) ensuring the Chemotherapy CRG is fully informed of clinical need, typically via the local Area Team. The pharmaceutical industry can support HCPs in the fulfilment of these roles, for example through timely provision of information on forthcoming treatments, dissemination of published data, support for local collection of outcome data, assistance for services considering the role of a new treatment and planning for its introduction, facilitation of disease-specific forums and the provision of non-promotional educational meetings. Groups of HCPs are likely to have a stronger voice than individuals, and it is hoped that pharmaceutical companies will be able to work in collaboration with each other to support consensus meetings, in which HCPs can contribute to the development of new treatment algorithms in an objective and timely manner.

## NHS/patient engagement

The ‘users’ of NHSE services have a key role to play in the decision-making process for the commissioning of new treatments and therapies. ‘Transforming Participation in Health and Care’ [16] embeds patient and public participation in the commissioning decision-making processes at all levels of the NHS, including CCGs. At a national and strategic level NHSE has demonstrated commitment to working with a wide range of patients and patient groups evidenced by the inclusion of patient and public voice (PPV) representatives in the different committees involved with the commissioning of specialised cancer services, including the CRGs and the Clinical Priorities Advisory Group (CPAG). CPAG is a national group which makes formal recommendations to the NHSE Board where commissioning decisions could result in a substantial change in service provision.

## Remaining uncertainties

Various uncertainties remain in the new NHS in England at the time of writing. The projected lifespan of the CDF is one example. It is not clear whether the CDF will have a role after April 2016, and there is concern over whether the fund will be adequate to meet demand [9]. Meanwhile, it can be anticipated that the Chemotherapy CRG will decide whether new drugs should be referred to the CDF panel or commissioned through an (interim) commissioning policy. It is unclear whether new drugs being proposed for addition to the national CDF list will need to offer extra/alternative value to those drugs already included. Given the current and predicted financial challenges, an answer to this question should become apparent early in the 2014‒2015 financial year.

Value-based pricing—a system for ensuring that the price paid for a new medicine reflects its value to the NHS—was due to come into effect shortly after the main tranche of reforms, but its development was slower than originally anticipated [2]. In 2013, the concept was amended to one of value-based assessment of treatments, and is now under the aegis of NICE [17]. Value-based assessment is expected to use a simple weighting system for burden of illness, with additional measures relating to the wider societal benefits of the medicine in question, and its particular value when patients approach the end of life. Public consultation on value-based assessment will continue until summer 2014, with a view to implementation from autumn 2014. Meanwhile, it is clear that the NHSE commissioning decision-making process is already taking account of the value of drugs in terms of improving patient outcomes, quality of care, resource utility and, therefore, affordability. Looking forward, the consideration of some of these parameters will be facilitated through the mandated SACT data-set collection.

The mechanism for national horizon scanning remains unclear, and will be a priority for the Chemotherapy CRG. It is likely that the Chemotherapy CRG will ask other NHS committees or groups to conduct this service for them. Oncology stakeholders agree that advance notification by the pharmaceutical industry of expected drug licensing is essential for all new products and indications, along with details of their potential to offer added value to patients, NHS services and society.

Also, at the time of writing, the government has just launched the Early Access to Medicines Scheme. Its remit is to ‘give patients with life-threatening or seriously debilitating conditions access to medicines that do not yet have a marketing authorisation when there is a defined unmet medical need’ [18]. This new initiative, and its likely impact on access to new SACTs, will no doubt be a key topic for discussion HCPs and pharmaceutical companies over the ensuing months.

## Conclusion

The NHS has been a focus of change and reform since its inception. However, many observers believe that the recent (and ongoing) overhaul in England is on an unprecedented scale. As new drugs are developed, their role in the NHS in England is expected to depend on the findings of a thorough assessment of the value they offer the patient, the NHS, and wider society in terms of clinical, financial and organisational benefits. HCPs have an opportunity to be at the heart of the decision-making process, whether as members of the CCGs or CRGs, or through participation in disease-specific forums, consensus development and data development and collection.

Chemotherapy and other SACTs (other than hormonal treatments) are now commissioned nationally, via NHSE and the Chemotherapy CRG. The CDF will survive until at least 2016, and is now under the auspices of the NHSE. As with other treatments, drug cost is not the sole deciding factor for the introduction of new SACTs—value is judged on a broader scale. Oncology stakeholders who wish to see access to a particular new treatment will have opportunities to affect commissioning decisions, ideally as part of a collaborative group. The pharmaceutical industry will also play an important role, through the timely provision of information to all stakeholders along the decision-making path.

## Figures and Tables

**Table 1.** NHS Outcomes Framework: the five domains and their possible implications for cancer commissioning [13].

Domain number	Key outcome	Possible implications for cancer commissioning (examples)
1	Preventing people from dying prematurely	Provision of treatments that maximise the likelihood of cure or extended survival
2	Enhancing quality of life for people with long-term conditions	Consideration of factors such as treatment side effects, and models of service delivery that minimise disruption to patients’ lifestyles
3	Helping people to recover from episodes of ill health or following injury	Consideration of treatments that can help patients retain independent living, and those that may reduce the likelihood of emergency admissions
4	Ensuring that people have a positive experience of care	Consideration of the needs of people approaching the end of life
5	Treating and caring for people in a safe environment and protecting them from avoidable harm	Consideration of treatment safety
